# Bis(4-{2-[4-(diethyl­amino)­phen­yl]ethen­yl}pyridine-κ*N*)diiodidozinc

**DOI:** 10.1107/S1600536813001736

**Published:** 2013-02-02

**Authors:** Cui-Yun Nie, Yu-Peng Tian

**Affiliations:** a Department of Chemistry, Anhui University, Hefei 230039, People’s Republic of China; b Key Laboratory of Functional Inorganic Materials, Chemistry, Hefei 230039, People’s Republic of China

## Abstract

In the title compound, [ZnI_2_(C_17_H_20_N_2_)_2_], the Zn^II^ atom is four-coordinated by two I atoms and the N atoms of two pyridine rings belonging to different ligands in a distorted tetra­hedral geometry. The coordinating pyridine rings are oriented in an almost perpendicular fashion, making a dihedral angle of 83.7 (5)°.

## Related literature

For the crystal structures of Zn complexes with related pyridine derivatives, see: Wang *et al.* (2012 ▶); Gao *et al.* (2009 ▶).
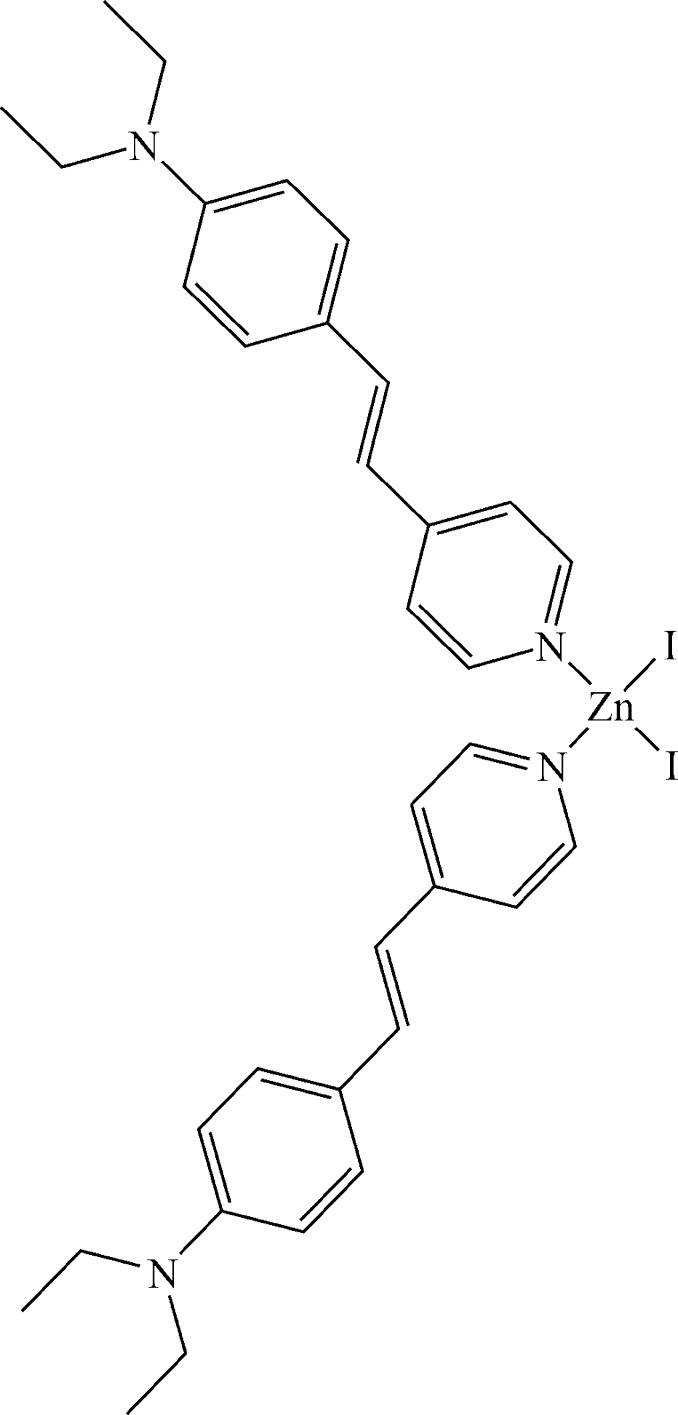


## Experimental

### 

#### Crystal data

[ZnI_2_(C_17_H_20_N_2_)_2_]
*M* *_r_* = 823.87
Monoclinic, 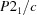

*a* = 13.724 (5) Å
*b* = 9.861 (8) Å
*c* = 27.742 (3) Å
β = 112.693 (12)°
*V* = 3464 (3) Å^3^
*Z* = 4
Mo *K*α radiation
μ = 2.52 mm^−1^
*T* = 293 K
0.31 × 0.23 × 0.22 mm

#### Data collection

Bruker SMART CCD area-detector diffractometer
Absorption correction: multi-scan (*SADABS*; Bruker, 2002 ▶) *T*_min_ = 0.509, *T*_max_ = 0.607
23970 measured reflections
6097 independent reflections
3568 reflections with *I* > 2σ(*I*)
*R*_int_ = 0.049

#### Refinement

*R*[*F* ^2^ > 2σ(*F* ^2^)] = 0.063
*wR*(*F* ^2^) = 0.197
*S* = 1.04
6097 reflections
374 parameters
305 restraints
H-atom parameters constrained
Δρ_max_ = 1.44 e Å^−3^
Δρ_min_ = −0.95 e Å^−3^

### 

Data collection: *SMART* (Bruker, 2002 ▶); cell refinement: *SAINT* (Bruker, 2002 ▶); data reduction: *SAINT*; program(s) used to solve structure: *SHELXTL* (Sheldrick, 2008 ▶); program(s) used to refine structure: *SHELXTL*; molecular graphics: *SHELXTL*; software used to prepare material for publication: *SHELXTL*.

## Supplementary Material

Crystal structure: contains datablock(s) I, global. DOI: 10.1107/S1600536813001736/lr2093sup1.cif

Structure factors: contains datablock(s) I. DOI: 10.1107/S1600536813001736/lr2093Isup2.hkl

Additional supplementary materials:  crystallographic information; 3D view; checkCIF report

## Figures and Tables

**Fig. 1.**
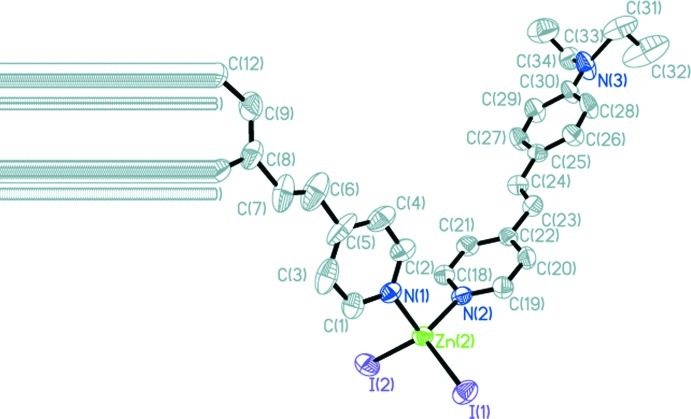
: The molecular structure of the title molecule(I) showing 30% probability displacement ellipsoids.

## supplementary crystallographic information

### Comment

In recent years, pyridine based materials have attracted considerable interests because of their significant applications in the areas of nonlinear optical (NLO), such as two-photon excited fluorescence (TPEF) microscopy, optical limiting. In addition, zinc complexes are particularly attractive and most studied for their biocompatibility (Wang *et al.*, 2012; Gao *et al.*, 2009). Herewith we present the structure of (I) which molecular structure is showed in Fig.1. The Zn^II^ is coordinated by the N atoms of two pyridine rings belonging to diferent ligands and two iodine atoms in a distorted tetrahedral geometry the Zn^II^ and with the coordinated pyridine moities oriented in an almost perpendicular fashion with a dihedral angle of 83.7 (5)°.

### Experimental

Fresh zinc iodide (0.32 g, 1 mmol) and the ligand (0.50 g, 2 mmol) were vigorously stirred in 15 ml of methanol until the solid phase had been completely dissolved, and then, the mixture was refluxed for 2 h. The reaction mixture was cooled to room temperature and filtered into a large test tube. Red brown needle crystals were obtained at room temperature after a week. Yield: 0.71 g (86%).

### Refinement

All hydrogen atoms were placed in geometrically idealized positions and constrained to ride on their parent atoms, with C—H = 0.93 Å and *U*_iso_(H) = 1.2 *U*_eq_.

### Crystal data

**Table:**

[ZnI_2_(C_17_H_20_N_2_)_2_]	*F*(000) = 1632
*M**_r_* = 823.87	*D*_x_ = 1.580 Mg m^−^^3^
Monoclinic, *P*2_1_/*c*	Mo *K*α radiation, λ = 0.71069 Å
Hall symbol: -P 2ybc	Cell parameters from 3863 reflections
*a* = 13.724 (5) Å	θ = 2.2–19.1°
*b* = 9.861 (8) Å	µ = 2.52 mm^−^^1^
*c* = 27.742 (3) Å	*T* = 293 K
β = 112.693 (12)°	Block, red
*V* = 3464 (3) Å^3^	0.31 × 0.23 × 0.22 mm
*Z* = 4	

### Data collection

**Table:**

Bruker SMART CCD area-detector diffractometer	6097 independent reflections
Radiation source: fine-focus sealed tube	3568 reflections with *I* > 2σ(*I*)
Graphite monochromator	*R*_int_ = 0.049
phi and ω scans	θ_max_ = 25.0°, θ_min_ = 1.6°
Absorption correction: multi-scan (*SADABS*; Bruker, 2002)	*h* = −16→16
*T*_min_ = 0.509, *T*_max_ = 0.607	*k* = −11→11
23970 measured reflections	*l* = −32→32

### Refinement

**Table:**

Refinement on *F*^2^	Primary atom site location: structure-invariant direct methods
Least-squares matrix: full	Secondary atom site location: difference Fourier map
*R*[*F*^2^ > 2σ(*F*^2^)] = 0.063	Hydrogen site location: inferred from neighbouring sites
*wR*(*F*^2^) = 0.197	H-atom parameters constrained
*S* = 1.04	*w* = 1/[σ^2^(*F*_o_^2^) + (0.0883*P*)^2^ + 10.7456*P*] where *P* = (*F*_o_^2^ + 2*F*_c_^2^)/3
6097 reflections	(Δ/σ)_max_ = 0.017
374 parameters	Δρ_max_ = 1.44 e Å^−^^3^
305 restraints	Δρ_min_ = −0.95 e Å^−^^3^

### Special details

**Table:**

Geometry. All e.s.d.'s (except the e.s.d. in the dihedral angle between two l.s. planes) are estimated using the full covariance matrix. The cell e.s.d.'s are taken into account individually in the estimation of e.s.d.'s in distances, angles and torsion angles; correlations between e.s.d.'s in cell parameters are only used when they are defined by crystal symmetry. An approximate (isotropic) treatment of cell e.s.d.'s is used for estimating e.s.d.'s involving l.s. planes.
Refinement. Refinement of *F*^2^ against ALL reflections. The weighted *R*-factor *wR* and goodness of fit *S* are based on *F*^2^, conventional *R*-factors *R* are based on *F*, with *F* set to zero for negative *F*^2^. The threshold expression of *F*^2^ > σ(*F*^2^) is used only for calculating *R*-factors(gt) *etc*. and is not relevant to the choice of reflections for refinement. *R*-factors based on *F*^2^ are statistically about twice as large as those based on *F*, and *R*- factors based on ALL data will be even larger.

### Fractional atomic coordinates and isotropic or equivalent isotropic displacement parameters (Å^2^)

**Table:**

	*x*	*y*	*z*	*U*_iso_*/*U*_eq_	
I1	−0.08626 (6)	0.88056 (8)	0.28023 (3)	0.0904 (3)	
I2	−0.14470 (6)	0.74792 (8)	0.41847 (3)	0.0923 (3)	
Zn2	−0.07433 (8)	0.69644 (10)	0.34668 (4)	0.0633 (3)	
N1	−0.1561 (6)	0.5329 (8)	0.3044 (3)	0.0682 (19)	
N2	0.0736 (6)	0.6182 (7)	0.3834 (3)	0.0634 (19)	
N3	0.7603 (9)	0.0057 (13)	0.5880 (5)	0.128 (3)	
N4	−0.6764 (8)	−0.2949 (11)	0.1114 (5)	0.109 (3)	
C1	−0.2622 (9)	0.5358 (13)	0.2838 (4)	0.092 (2)	
H1	−0.2971	0.6131	0.2879	0.110*	
C2	−0.1121 (10)	0.4205 (11)	0.2965 (4)	0.087 (2)	
H2	−0.0387	0.4174	0.3099	0.104*	
C3	−0.3218 (10)	0.4242 (13)	0.2560 (4)	0.097 (2)	
H3	−0.3952	0.4289	0.2415	0.117*	
C4	−0.1640 (10)	0.3098 (12)	0.2709 (4)	0.095 (2)	
H4	−0.1260	0.2351	0.2673	0.114*	
C5	−0.2702 (11)	0.3058 (12)	0.2504 (5)	0.098 (2)	
C6	−0.3144 (9)	0.1787 (12)	0.2286 (4)	0.106 (2)	
H6	−0.2692	0.1044	0.2358	0.128*	
C7	−0.4166 (8)	0.1580 (12)	0.1984 (4)	0.099 (2)	
H7	−0.4585	0.2355	0.1901	0.119*	
C8	−0.4705 (9)	0.0341 (11)	0.1772 (5)	0.092 (2)	
C9	−0.4359 (9)	−0.1008 (12)	0.1869 (5)	0.095 (2)	
H9	−0.3662	−0.1185	0.2087	0.114*	
C10	−0.5727 (9)	0.0500 (12)	0.1444 (5)	0.093 (2)	
H10	−0.5982	0.1381	0.1367	0.111*	
C11	−0.6412 (9)	−0.0537 (11)	0.1217 (5)	0.089 (2)	
H11	−0.7099	−0.0341	0.0990	0.106*	
C12	−0.5036 (9)	−0.2085 (12)	0.1644 (5)	0.092 (2)	
H12	−0.4778	−0.2967	0.1712	0.111*	
C13	−0.6097 (8)	−0.1874 (12)	0.1319 (5)	0.086 (2)	
C14	−0.7856 (11)	−0.2734 (15)	0.0712 (6)	0.133 (4)	
H14A	−0.7844	−0.1988	0.0487	0.160*	
H14B	−0.8076	−0.3542	0.0497	0.160*	
C15	−0.8634 (12)	−0.2437 (17)	0.0942 (7)	0.155 (5)	
H15A	−0.8537	−0.3053	0.1225	0.233*	
H15B	−0.9334	−0.2541	0.0680	0.233*	
H15C	−0.8540	−0.1523	0.1071	0.233*	
C16	−0.6404 (10)	−0.4364 (14)	0.1182 (5)	0.116 (3)	
H16A	−0.5705	−0.4418	0.1173	0.140*	
H16B	−0.6879	−0.4911	0.0898	0.140*	
C17	−0.6373 (11)	−0.4898 (15)	0.1688 (5)	0.129 (4)	
H17A	−0.5780	−0.4516	0.1969	0.194*	
H17B	−0.6307	−0.5868	0.1693	0.194*	
H17C	−0.7013	−0.4655	0.1730	0.194*	
C18	0.0945 (8)	0.5466 (10)	0.4271 (4)	0.075 (2)	
H18	0.0434	0.5395	0.4413	0.090*	
C19	0.1497 (7)	0.6285 (10)	0.3646 (4)	0.075 (2)	
H19	0.1373	0.6817	0.3351	0.090*	
C20	0.2454 (7)	0.5636 (10)	0.3872 (4)	0.0739 (18)	
H20	0.2948	0.5703	0.3719	0.089*	
C21	0.1918 (8)	0.4819 (10)	0.4519 (4)	0.0741 (18)	
H21	0.2043	0.4334	0.4825	0.089*	
C22	0.2689 (7)	0.4886 (10)	0.4323 (4)	0.0712 (16)	
C23	0.3707 (8)	0.4164 (10)	0.4555 (4)	0.0734 (18)	
H23	0.4181	0.4263	0.4392	0.088*	
C24	0.3997 (8)	0.3399 (10)	0.4970 (4)	0.0740 (18)	
H24	0.3551	0.3402	0.5151	0.089*	
C25	0.4923 (8)	0.2545 (11)	0.5185 (4)	0.0773 (17)	
C26	0.5615 (9)	0.2317 (11)	0.4953 (4)	0.0835 (18)	
H26	0.5499	0.2723	0.4633	0.100*	
C27	0.5140 (8)	0.1901 (12)	0.5656 (4)	0.0846 (19)	
H27	0.4676	0.2027	0.5823	0.101*	
C28	0.6486 (8)	0.1497 (12)	0.5181 (4)	0.0881 (19)	
H28	0.6944	0.1384	0.5010	0.106*	
C29	0.5997 (8)	0.1090 (12)	0.5889 (4)	0.0896 (19)	
H29	0.6108	0.0700	0.6211	0.108*	
C30	0.6713 (9)	0.0831 (13)	0.5653 (5)	0.0906 (19)	
C33	0.7943 (11)	−0.0354 (15)	0.6460 (6)	0.131 (3)	
H33A	0.8699	−0.0503	0.6624	0.157*	
H33B	0.7740	0.0327	0.6656	0.157*	
C34	0.7347 (12)	−0.1634 (16)	0.6417 (7)	0.152 (5)	
H34A	0.6606	−0.1465	0.6235	0.229*	
H34B	0.7474	−0.1974	0.6760	0.229*	
H34C	0.7576	−0.2292	0.6228	0.229*	
C31	0.8072 (11)	−0.0722 (15)	0.5527 (6)	0.142 (3)	
H31A	0.8343	−0.1626	0.5640	0.171*	
H31B	0.7651	−0.0681	0.5155	0.171*	
C32	0.8949 (11)	0.0225 (17)	0.5633 (7)	0.158 (4)	
H32A	0.8684	0.1079	0.5469	0.238*	
H32B	0.9440	−0.0136	0.5497	0.238*	
H32C	0.9300	0.0353	0.6004	0.238*	

### Atomic displacement parameters (Å^2^)

**Table:**

	*U*^11^	*U*^22^	*U*^33^	*U*^12^	*U*^13^	*U*^23^
I1	0.0763 (5)	0.0786 (5)	0.0931 (6)	−0.0070 (4)	0.0071 (4)	0.0243 (4)
I2	0.0928 (6)	0.0935 (6)	0.0923 (6)	0.0125 (4)	0.0375 (4)	−0.0147 (4)
Zn2	0.0576 (6)	0.0539 (6)	0.0683 (7)	−0.0019 (5)	0.0132 (5)	−0.0030 (5)
N1	0.078 (4)	0.063 (4)	0.064 (4)	−0.015 (3)	0.028 (3)	−0.006 (3)
N2	0.064 (5)	0.051 (4)	0.068 (5)	0.000 (3)	0.017 (4)	0.004 (4)
N3	0.105 (5)	0.148 (6)	0.129 (5)	0.035 (4)	0.043 (4)	0.051 (5)
N4	0.090 (5)	0.093 (5)	0.141 (7)	−0.036 (5)	0.039 (5)	−0.038 (6)
C1	0.096 (5)	0.100 (5)	0.079 (5)	−0.029 (4)	0.032 (4)	−0.005 (4)
C2	0.103 (4)	0.078 (4)	0.078 (4)	−0.017 (3)	0.033 (4)	−0.010 (4)
C3	0.102 (4)	0.105 (4)	0.081 (4)	−0.035 (4)	0.031 (4)	−0.004 (4)
C4	0.116 (4)	0.086 (4)	0.079 (4)	−0.027 (3)	0.035 (4)	−0.012 (4)
C5	0.116 (4)	0.098 (4)	0.080 (4)	−0.038 (3)	0.037 (3)	−0.008 (3)
C6	0.116 (4)	0.108 (4)	0.092 (4)	−0.038 (4)	0.036 (4)	−0.013 (4)
C7	0.105 (4)	0.104 (4)	0.094 (4)	−0.035 (4)	0.044 (4)	−0.017 (4)
C8	0.091 (4)	0.092 (3)	0.098 (4)	−0.028 (3)	0.041 (3)	−0.018 (4)
C9	0.079 (4)	0.097 (4)	0.105 (5)	−0.024 (3)	0.032 (4)	−0.021 (4)
C10	0.092 (4)	0.082 (4)	0.104 (5)	−0.021 (3)	0.039 (4)	−0.019 (4)
C11	0.083 (4)	0.078 (4)	0.103 (5)	−0.015 (3)	0.034 (4)	−0.020 (4)
C12	0.075 (4)	0.088 (4)	0.109 (5)	−0.017 (3)	0.029 (4)	−0.021 (4)
C13	0.075 (4)	0.079 (4)	0.102 (5)	−0.017 (4)	0.032 (4)	−0.024 (4)
C14	0.112 (7)	0.117 (7)	0.166 (9)	−0.033 (6)	0.048 (6)	−0.035 (7)
C15	0.141 (9)	0.151 (10)	0.179 (11)	−0.027 (8)	0.068 (7)	−0.037 (9)
C16	0.105 (6)	0.110 (6)	0.131 (7)	−0.035 (5)	0.042 (6)	−0.040 (6)
C17	0.126 (8)	0.130 (9)	0.131 (8)	−0.034 (7)	0.048 (8)	−0.041 (7)
C18	0.070 (4)	0.073 (5)	0.081 (5)	0.008 (4)	0.029 (4)	0.013 (4)
C19	0.064 (4)	0.081 (5)	0.077 (5)	0.006 (4)	0.023 (4)	0.017 (4)
C20	0.062 (4)	0.080 (4)	0.079 (4)	0.005 (3)	0.027 (3)	0.013 (3)
C21	0.071 (3)	0.073 (4)	0.076 (4)	0.009 (3)	0.027 (3)	0.015 (3)
C22	0.064 (3)	0.071 (3)	0.076 (3)	0.005 (3)	0.023 (3)	0.010 (3)
C23	0.067 (3)	0.074 (4)	0.075 (4)	0.006 (3)	0.023 (3)	0.009 (3)
C24	0.069 (3)	0.078 (4)	0.073 (4)	0.008 (3)	0.025 (3)	0.009 (3)
C25	0.073 (3)	0.086 (4)	0.075 (3)	0.015 (3)	0.031 (3)	0.016 (3)
C26	0.082 (4)	0.097 (4)	0.076 (4)	0.022 (3)	0.036 (3)	0.023 (3)
C27	0.079 (4)	0.102 (4)	0.079 (4)	0.021 (3)	0.039 (3)	0.022 (3)
C28	0.082 (4)	0.109 (5)	0.082 (4)	0.026 (3)	0.042 (3)	0.029 (4)
C29	0.085 (4)	0.110 (5)	0.082 (4)	0.026 (3)	0.041 (3)	0.030 (4)
C30	0.085 (4)	0.112 (4)	0.086 (4)	0.029 (3)	0.044 (3)	0.033 (3)
C33	0.108 (6)	0.146 (7)	0.134 (5)	0.029 (5)	0.040 (5)	0.045 (6)
C34	0.132 (9)	0.147 (9)	0.154 (9)	0.015 (7)	0.029 (8)	0.027 (9)
C31	0.120 (6)	0.158 (7)	0.136 (6)	0.032 (5)	0.034 (5)	0.039 (6)
C32	0.129 (7)	0.164 (8)	0.149 (7)	0.021 (5)	0.017 (6)	0.042 (6)

### Geometric parameters (Å, º)

**Table:**

I1—Zn2	2.5473 (17)	C15—H15C	0.9600
I2—Zn2	2.5770 (14)	C16—C17	1.486 (9)
Zn2—N2	2.039 (7)	C16—H16A	0.9700
Zn2—N1	2.054 (8)	C16—H16B	0.9700
N1—C2	1.321 (13)	C17—H17A	0.9600
N1—C1	1.344 (13)	C17—H17B	0.9600
N2—C18	1.335 (12)	C17—H17C	0.9600
N2—C19	1.340 (12)	C18—C21	1.397 (13)
N3—C30	1.371 (14)	C18—H18	0.9300
N3—C33	1.546 (17)	C19—C20	1.376 (13)
N3—C31	1.564 (9)	C19—H19	0.9300
N4—C13	1.373 (13)	C20—C22	1.381 (13)
N4—C16	1.468 (16)	C20—H20	0.9300
N4—C14	1.499 (17)	C21—C22	1.366 (13)
C1—C3	1.410 (15)	C21—H21	0.9300
C1—H1	0.9300	C22—C23	1.477 (13)
C2—C4	1.347 (14)	C23—C24	1.304 (13)
C2—H2	0.9300	C23—H23	0.9300
C3—C5	1.405 (17)	C24—C25	1.448 (13)
C3—H3	0.9300	C24—H24	0.9300
C4—C5	1.345 (17)	C25—C26	1.355 (14)
C4—H4	0.9300	C25—C27	1.378 (14)
C5—C6	1.422 (9)	C26—C28	1.378 (14)
C6—C7	1.344 (8)	C26—H26	0.9300
C6—H6	0.9300	C27—C29	1.362 (14)
C7—C8	1.431 (9)	C27—H27	0.9300
C7—H7	0.9300	C28—C30	1.389 (14)
C8—C10	1.354 (15)	C28—H28	0.9300
C8—C9	1.404 (16)	C29—C30	1.398 (14)
C9—C12	1.390 (14)	C29—H29	0.9300
C9—H9	0.9300	C33—C34	1.484 (9)
C10—C11	1.368 (14)	C33—H33A	0.9700
C10—H10	0.9300	C33—H33B	0.9700
C11—C13	1.382 (15)	C34—H34A	0.9600
C11—H11	0.9300	C34—H34B	0.9600
C12—C13	1.402 (15)	C34—H34C	0.9600
C12—H12	0.9300	C31—C32	1.461 (9)
C14—C15	1.469 (9)	C31—H31A	0.9702
C14—H14A	0.9700	C31—H31B	0.9698
C14—H14B	0.9700	C32—H32A	0.9600
C15—H15A	0.9600	C32—H32B	0.9600
C15—H15B	0.9600	C32—H32C	0.9600
			
N2—Zn2—N1	102.0 (3)	N4—C16—H16B	109.6
N2—Zn2—I1	113.9 (2)	C17—C16—H16B	109.6
N1—Zn2—I1	106.1 (2)	H16A—C16—H16B	108.2
N2—Zn2—I2	106.6 (2)	C16—C17—H17A	109.5
N1—Zn2—I2	108.1 (2)	C16—C17—H17B	109.5
I1—Zn2—I2	118.71 (6)	H17A—C17—H17B	109.5
C2—N1—C1	115.9 (9)	C16—C17—H17C	109.5
C2—N1—Zn2	124.7 (7)	H17A—C17—H17C	109.5
C1—N1—Zn2	119.4 (8)	H17B—C17—H17C	109.5
C18—N2—C19	117.8 (8)	N2—C18—C21	121.3 (9)
C18—N2—Zn2	118.6 (7)	N2—C18—H18	119.3
C19—N2—Zn2	123.5 (6)	C21—C18—H18	119.3
C30—N3—C33	118.8 (11)	N2—C19—C20	122.6 (9)
C30—N3—C31	119.6 (11)	N2—C19—H19	118.7
C33—N3—C31	119.4 (11)	C20—C19—H19	118.7
C13—N4—C16	122.9 (11)	C19—C20—C22	120.6 (9)
C13—N4—C14	121.0 (11)	C19—C20—H20	119.7
C16—N4—C14	114.9 (10)	C22—C20—H20	119.7
N1—C1—C3	121.4 (12)	C22—C21—C18	121.4 (9)
N1—C1—H1	119.3	C22—C21—H21	119.3
C3—C1—H1	119.3	C18—C21—H21	119.3
N1—C2—C4	125.9 (12)	C21—C22—C20	116.2 (9)
N1—C2—H2	117.1	C21—C22—C23	123.4 (9)
C4—C2—H2	117.1	C20—C22—C23	120.3 (9)
C5—C3—C1	119.9 (12)	C24—C23—C22	125.4 (10)
C5—C3—H3	120.1	C24—C23—H23	117.3
C1—C3—H3	120.1	C22—C23—H23	117.3
C5—C4—C2	120.8 (13)	C23—C24—C25	128.3 (10)
C5—C4—H4	119.6	C23—C24—H24	115.8
C2—C4—H4	119.6	C25—C24—H24	115.8
C4—C5—C3	116.1 (10)	C26—C25—C27	116.0 (9)
C4—C5—C6	114.8 (12)	C26—C25—C24	124.5 (10)
C3—C5—C6	129.0 (12)	C27—C25—C24	119.5 (9)
C7—C6—C5	124.7 (12)	C25—C26—C28	121.5 (10)
C7—C6—H6	117.6	C25—C26—H26	119.2
C5—C6—H6	117.6	C28—C26—H26	119.2
C6—C7—C8	129.4 (12)	C29—C27—C25	123.5 (10)
C6—C7—H7	115.3	C29—C27—H27	118.3
C8—C7—H7	115.3	C25—C27—H27	118.3
C10—C8—C9	115.1 (10)	C26—C28—C30	123.3 (10)
C10—C8—C7	114.5 (11)	C26—C28—H28	118.3
C9—C8—C7	130.3 (12)	C30—C28—H28	118.3
C12—C9—C8	121.4 (11)	C27—C29—C30	121.3 (10)
C12—C9—H9	119.3	C27—C29—H29	119.4
C8—C9—H9	119.3	C30—C29—H29	119.4
C8—C10—C11	124.9 (12)	N3—C30—C28	122.3 (10)
C8—C10—H10	117.5	N3—C30—C29	123.3 (10)
C11—C10—H10	117.5	C28—C30—C29	114.3 (10)
C10—C11—C13	120.9 (11)	C34—C33—N3	101.7 (13)
C10—C11—H11	119.5	C34—C33—H33A	111.4
C13—C11—H11	119.5	N3—C33—H33A	111.4
C9—C12—C13	121.6 (12)	C34—C33—H33B	111.4
C9—C12—H12	119.2	N3—C33—H33B	111.4
C13—C12—H12	119.2	H33A—C33—H33B	109.3
N4—C13—C11	123.1 (11)	C33—C34—H34A	109.5
N4—C13—C12	120.9 (11)	C33—C34—H34B	109.5
C11—C13—C12	116.0 (10)	H34A—C34—H34B	109.5
C15—C14—N4	113.0 (14)	C33—C34—H34C	109.5
C15—C14—H14A	109.0	H34A—C34—H34C	109.5
N4—C14—H14A	109.0	H34B—C34—H34C	109.5
C15—C14—H14B	109.0	C32—C31—N3	93.8 (12)
N4—C14—H14B	109.0	C32—C31—H31A	109.8
H14A—C14—H14B	107.8	N3—C31—H31A	116.6
C14—C15—H15A	109.5	C32—C31—H31B	107.1
C14—C15—H15B	109.5	N3—C31—H31B	115.2
H15A—C15—H15B	109.5	H31A—C31—H31B	112.3
C14—C15—H15C	109.5	C31—C32—H32A	109.5
H15A—C15—H15C	109.5	C31—C32—H32B	109.4
H15B—C15—H15C	109.5	H32A—C32—H32B	109.5
N4—C16—C17	110.1 (12)	C31—C32—H32C	109.5
N4—C16—H16A	109.6	H32A—C32—H32C	109.5
C17—C16—H16A	109.6	H32B—C32—H32C	109.5
			
N2—Zn2—N1—C2	−12.9 (9)	C9—C12—C13—N4	−177.8 (12)
I1—Zn2—N1—C2	106.6 (8)	C9—C12—C13—C11	2.8 (18)
I2—Zn2—N1—C2	−125.0 (8)	C13—N4—C14—C15	−86.0 (16)
N2—Zn2—N1—C1	165.5 (7)	C16—N4—C14—C15	106.3 (14)
I1—Zn2—N1—C1	−75.0 (7)	C13—N4—C16—C17	84.5 (15)
I2—Zn2—N1—C1	53.3 (8)	C14—N4—C16—C17	−108.1 (13)
N1—Zn2—N2—C18	−82.1 (7)	C19—N2—C18—C21	−1.3 (14)
I1—Zn2—N2—C18	164.0 (6)	Zn2—N2—C18—C21	175.2 (7)
I2—Zn2—N2—C18	31.1 (7)	C18—N2—C19—C20	3.0 (14)
N1—Zn2—N2—C19	94.1 (8)	Zn2—N2—C19—C20	−173.3 (8)
I1—Zn2—N2—C19	−19.8 (8)	N2—C19—C20—C22	−2.9 (16)
I2—Zn2—N2—C19	−152.6 (7)	N2—C18—C21—C22	−0.5 (16)
C2—N1—C1—C3	0.7 (15)	C18—C21—C22—C20	0.7 (15)
Zn2—N1—C1—C3	−177.8 (8)	C18—C21—C22—C23	−176.7 (9)
C1—N1—C2—C4	−1.4 (16)	C19—C20—C22—C21	0.9 (15)
Zn2—N1—C2—C4	177.0 (9)	C19—C20—C22—C23	178.4 (10)
N1—C1—C3—C5	1.2 (17)	C21—C22—C23—C24	−0.6 (17)
N1—C2—C4—C5	0.0 (19)	C20—C22—C23—C24	−178.0 (11)
C2—C4—C5—C3	2.0 (18)	C22—C23—C24—C25	172.4 (10)
C2—C4—C5—C6	−174.6 (11)	C23—C24—C25—C26	−5.9 (19)
C1—C3—C5—C4	−2.6 (17)	C23—C24—C25—C27	174.3 (11)
C1—C3—C5—C6	173.5 (11)	C27—C25—C26—C28	−0.6 (18)
C4—C5—C6—C7	−167.9 (12)	C24—C25—C26—C28	179.6 (11)
C3—C5—C6—C7	16 (2)	C26—C25—C27—C29	0.8 (19)
C5—C6—C7—C8	−175.7 (12)	C24—C25—C27—C29	−179.4 (11)
C6—C7—C8—C10	−174.5 (13)	C25—C26—C28—C30	1 (2)
C6—C7—C8—C9	9 (2)	C25—C27—C29—C30	−1 (2)
C10—C8—C9—C12	−0.8 (18)	C33—N3—C30—C28	165.0 (13)
C7—C8—C9—C12	175.8 (12)	C31—N3—C30—C28	−32 (2)
C9—C8—C10—C11	0.8 (19)	C33—N3—C30—C29	−11 (2)
C7—C8—C10—C11	−176.4 (11)	C31—N3—C30—C29	152.4 (13)
C8—C10—C11—C13	1.1 (19)	C26—C28—C30—N3	−177.8 (13)
C8—C9—C12—C13	−1.0 (19)	C26—C28—C30—C29	−2 (2)
C16—N4—C13—C11	174.0 (12)	C27—C29—C30—N3	177.9 (13)
C14—N4—C13—C11	7.3 (18)	C27—C29—C30—C28	1.8 (19)
C16—N4—C13—C12	−5.3 (18)	C30—N3—C33—C34	88.2 (16)
C14—N4—C13—C12	−172.0 (12)	C31—N3—C33—C34	−75.0 (16)
C10—C11—C13—N4	177.8 (12)	C30—N3—C31—C32	104.9 (14)
C10—C11—C13—C12	−2.9 (17)	C33—N3—C31—C32	−92.0 (14)
